# Does dental health of 6-year-olds reflect the reform of the Israeli dental care system?

**DOI:** 10.1186/s13584-016-0086-3

**Published:** 2016-10-05

**Authors:** Lena Natapov, Avi Sasson, Shlomo P. Zusman

**Affiliations:** 1 Division of Dental Health, Ministry of Health, 37 Yirmiyahu Street, Jerusalem, Israel; 2 Association of Public Health, Jerusalem, Israel

**Keywords:** Tooth decay, Children, Prevention of dental diseases, Morbidity, Reform, National health insurance law, Public health policy

## Abstract

**Background:**

The National health insurance law enacted in 1995 did not include dental care in its basket of services. Dental care for children was first included in 2010, initially up till 8 years of age. The eligibility age rose to 12 years in 2013.

The dental survey of 6 year-olds in 2007 found that the average of decayed, missing and filled teeth index (dmft) was 3.31 and 35 % of children were caries free. The current cross sectional survey of dental health for 6 year-olds was conducted as a comparison to the pre-reform status.

**Methods:**

Twenty-three local authorities were randomly selected nationwide. Two Grade 1 classes were randomly chosen in each. The city of Jerusalem was also included in the survey because of its size. The children were examined according to the WHO Oral Health Survey Methods 4th ed protocol. The dental caries index for deciduous teeth (dmft: decayed, missing, filled teeth) was calculated.

**Results:**

One thousand two hundred ten children were examined. 61.7 % of the children suffered from dental decay and only 38.3 % were caries free. The mean dmft was 2.56; d = 1.41 (teeth with untreated caries), f = 1.15 (teeth damaged by decay and restored), virtually none were missing due to caries. Dental caries prevalence was rather consistent, an average of over 2 teeth affected per child. Although there is no major change in comparison to former surveys, there is more treated than untreated disease. In the present survey the f component is higher than in the past, especially in the Jewish sector where it is the main component. It is still lower in the Arab sector.

**Conclusions:**

Although the level of dental disease remained rather constant, an increase in the treatment component was observed. In order to reduce caries prevalence, preventive measures such as school dental services and drinking water fluoridation should be extended and continued. Primary preventive dental services should be established for children from birth, with an emphasis on primary health care and educational settings, such as family health centers and kindergartens.

## Background

The National Health Insurance Law (NHIL).was enacted in 1994 and set the state’s responsibility for health care provision to all citizens. The health care is provided by four Health Maintenance Organizations (HMO). Dental care was not included in the basket of health care services, only oral and maxillo-facial surgery, in cases of trauma and tumors, and limited care to special groups such as oncology patients.

Dental care policy in Israel underwent a substantial change in the past few years.

Until 2010, only School Dental Services (SDS), financed by the state and provided by local authorities, offered preventative and conservative dental care to a relatively small percentage of primary school students. Generally, dental care was paid for primarily out of pocket, constituting about 10 % of national health expenditure, higher than in many Western countries. Yet, most industrialized countries have better dental health. It was a market failure; the high costs did not translate into a better oral health for Israeli citizens.

In 2007, for the first time, dental care for children was included in additional health services (AHS) plans offered by the HMO’s to their members.

The major change occurred in 2010 with inclusion of dental care for children into the National health Insurance Law (NHIL). The government approved the plan, proposed by Deputy Health Minister Yaakov Litzman, to finance dental care for children. In 2010 it diverted NIS 65 million from the budget for updating the basket of services of the NIHL to finance part of the inclusion of dental care.

At the beginning, children from birth to 8-years were entitled to the treatments, then the eligibility age went up gradually and, in 2013, children were covered by national dental health insurance up to age 12.

Additionally, the School Dental Service (SDS) budget was tripled in 2010 to NIS 30 million, acknowledging the state’s responsibility for community-based primary oral health services for all schoolchildren. This eliminated the need for parents and local authorities’ participation in SDS financing and reduced the barrier for local authorities to join it.

The change in patterns of dental disease, its severity and distribution and treatment level is of special interest after the reform.

A national dental survey among primary school children was conducted in Israel in 1990 [1]; the average dmft was 2.7, 41 % of children were caries free. Since then no national study has been conducted in this age group.

In 2007 a survey among 5 year-olds in 28 of 70 local authorities providing School Dental Services found that 64.7 % of children suffered from tooth decay, with an average dmft (dmft: decayed, missing, filled teeth) of 3.31, which consisted of d = 2.71, f = 0.49, m = 0.11.

A national survey of 12-year-olds carried out in 2002 [2] found that children had on average 1.66 affected permanent teeth, with approximately 46 % caries free. Unfortunately, 6-year-olds were not included in this survey.

In 2011 The Israel National Institute for Health Policy (NIHP) approved a grant for assessing the oral health status of 6-year-old children in view of dental care reforms.

The aim of the current study was to collect up-to-date epidemiological data on the dental health of 6-year-olds in Israel to assess if dental reform is already reflected in oral health of young schoolchildren.

## Methods

After approval of the Ministry of Health’s Ethics Committee (No. 11/2013), a random, stratified, cluster sample was selected.

Initially local authorities (LA) were divided into 3 strata:

A. Urban over 10,000 people
B. Urban under 10,000 people
C. Rural

Authorities were randomly chosen from the Central Bureau of Statistics’ list: 18 from strata A and 2 from strata B and C, according to their proportions within the Israeli population.

Additionally, Jerusalem was included in the sample because of its size.

In the second stage, two schools were randomly chosen in each LA. Finally, one first grade in each school was randomly chosen. In Jerusalem 6 classes were chosen, two of them from the Arab sector.

In Kibbutz Ginosar, where there is only one school, two classes were sampled from the same school.

The sample size was similar to samples of former National surveys, large enough to be valid for the different population groups.

### Examination method

The children were examined according to the WHO Oral Health Survey Methods Ed.4 [3]. The child was examined sitting, facing a natural light source, using a dental mirror only. The data recorded: dmft (decayed, missing, filled teeth) which measures caries experience in primary dentition.

The d component of the index shows the number of teeth affected by caries and untreated. The f component refers to teeth affected by decay and restored. The m refers to missing teeth due to dental caries.

### Training and calibration of surveyors

The survey was conducted by 8 experienced dentists working for the School Dental Services who participated in a calibration exercise conducted by an experienced senior researcher until a Cohen’s Kappa of 0.7 or higher was achieved.

### SES

Social Security classifies LAs by Socio Economic Level of the population [4]. 1 is the lowest and 10 the highest. In our study, they were clustered in 3 groups: 1-4 defined as low SES, 5-7 as medium and 8–10 as high.

### Statistical analysis

The findings were entered and calculated using an SPSSWIN + application. dmft index was calculated, as well as the percentage of children without caries. Mann-Whitney test was used to analyze differences between groups, *p* < 0.05 was regarded as significant.

## Results

One thousand two hundred ten students in first grade (average age 6 years) in 23 authorities underwent a dental examination during the 2014–2015 school year. 61.7 % of the children suffered from dental decay and only 38.3 % were free of caries. The mean dmft was 2.56; d = 1.41 (teeth with untreated caries), f = 1.15 (teeth damaged by decay and restored) (Table 1).

**Table 1:** dmft by gender and sector

	Caries free	d	m	f	dmft (sd)
Total	38.3 %	1.41	0.00	1.15	2.56 (2.92)
Gender					
Boys	36.2 %	1.39	0.00	1.33	2.72 (3.04)
Girls	38.8 %	1.43	0.00	0.94	2.37 (2.76)
Sector					
Jews	44.0 %	0.78	0.00	1.24	2.02 (2.60)
Arabs	21.6 %	2.90	0.00	0.95	3.85 (3.23)

### dmft by gender

Six hundred fifty-eight boys and 552 girls were examined. Boys had more tooth decay (dmft = 2.72 compared with 2.37 among girls, *p* < 0.1), the percentage of boys without tooth decay was lower than that of girls (36.2 % vs. 38.8 %). The treatment index (f/dmft) was higher in the boys (0.49) than in the girls (0.40) (*p* < 0.002) and the number of missing teeth as a result of decay was identical in both groups.

### dmft by ethnic group

Eight hundred fifty-three students from Jewish sector 357 students from Arab sector were examined. The dmft index among Arab children was significantly higher (3.85 vs. 2.02, *p* < 0.001). The percentage of children who were affected by tooth decay was high in both groups, especially in the Arab sector (78.4 % versus 54.7 %).

The main component of the dmft index in the Arab sector was d component representing the non-treated dental disease (2.90 compared to 0.78 in the Jewish sector).

Among Jewish children, more teeth were restored than decayed and not treated (f component of the dmft index was higher than d). The treatment index among Jewish children was 61 %, much higher than that of Arabs with only 25 % (*p* < 0.001), (f = 1.24 compared with 0.95).

### dmft by SES

An inverse correlation between socioeconomic strata and the prevalence of caries was found: the higher the socio-economic level, the lower the level of dental disease recorded. The differences between the percentage of caries free and mean dmft were statistically significant (*p* < 0.003) for all three clusters (cluster 1 vs. 2, cluster 2 vs. versus 3, cluster 1 vs. 3). The d component is larger than the f component only in the low SES, in the middle group and the high group, it is much less (Table 2).

**Table 2:** dmft by SES group

SES group	n	Caries free	d	m	f	dmft (s.d)
1–4	703	27.30 %	1.97	0.00	1.38	3.35 (3.14)
5–7	307	48.20 %	0.80	0.00	0.94	1.74 (2.37)
8–10	200	61.50 %	0.34	0.00	0.71	1.05 (1.73)

## Discussion

On average more than 2 teeth were affected per child (dmft = 2.56) and only 38.3 % of the children never experienced the disease.

Boys showed a higher prevalence than girls and this distribution was expected considering the dental health survey’s findings in Israel [5].

Dental caries is a lifestyle – related disease, more prevalent in low SES, education and income groups [6].

In 2007 [5], 1647 5-year-olds were surveyed from 28 local authorities (14 Jewish and14 Arab) and 35.3 % of the children were caries free. The mean dmft ± s.d was 3.31 ± 3.7, dt = 2.71 ± 3.48, mt = 0.11 ± 0.61, ft = 0.49 ± 1.41. Arab children had a lower percentage of caries free, higher dt, mt and dmft (14.9 %, 4.85 ± 3.83, 0.14 ± 0.58 and 4.38 ± 3.83 respectively), but lower ft = 0.33 ± 1.14) than Jewish which were 49.9 % caries free, 2.21 ± 3.15, 1.53 ± 2.66, 0.08 ± 0.63 and 0.6 ± 1.57 respectively.

The national survey of 12-year-olds conducted in 2002 [2] indicated a relatively high prevalence of the disease in Israel compared to other Western countries. The present survey findings are also higher than in other Western countries. In Denmark [7] 86 % were caries free, Sweden [8] 77 %, Wales [9] 59 %, Portugal [10] 55 % and in Austria [11], 52 %. Privately funded dental treatment is a barrier to care for those with low SES, and dental disease is more prevalent among children with low SES. That is the reason that “dental systems relying on public coverage seem to show lower inequalities in their use, thus confirming the potential benefits of such systems” [12]. Hence, we had expected to find a benefit from the reform, with less untreated and more treated disease than in pre-reform surveys.

Whilst there is no major change in total dmft compared to former survey, for the first time we found far more treated then untreated disease: In the present survey the f component is much higher than the d, especially in the Jewish sector where it is the main component.

The change is less dramatic in the Arab sector, but still improved [13].

Research into the utilization pattern and children’s experience in dental care following the reform, published in 2014 [14] arrived at the same conclusion – Arabs with low SES have less treated disease that Jews with low SES in the Health Maintenance Organization (HMO) clinics and prefer to go to private dentists.

We cannot rule out the possibility that additional factors, other than the 2010 reform, may have contributed to the changes observed. As the reform was nation-wide, no control group was available to help tease out the effects of the reform.

### Public policy issues

For the first time during the last few decades, the treated disease component (f) of the dmft index exceeded the decay (untreated component) (d), although this change was observed mainly in the Jewish sector. Further research of dental health related attitudes and behavior among the Arab sector is needed to understand the differences in dental health and dental treatment levels despite the universal entitlement for free treatment. The prevalence of dental caries among 5–6 year olds in Israel remained rather constant and further measures are required to lower it. It is important that community based oral health promoting programs receive the same priority as general health and clinical care. This was not so prior to 2010.

Dental disease amongst children could be prevented by extending preventative community services for children to younger ages and operating in community centers such as family health centers and kindergartens. Family Health Centers (Tipat Halav) in Israel are country-wide, accessible settings, providing free pre and post natal care. They are almost universally visited by mothers of newborns and toddlers. The public health nurses in these settings provide preventative health education and therefore are in an excellent position to provide preventative dental health advice to the mothers and are ready to do so [15, 16]. Recent research in Scotland showed the cost effectiveness of their “Childsmile” program which included daily supervised brushing in nursery settings. The program demonstrated substantial savings for the NHS [17]. A similar program was successfully implemented in 700 Israeli kindergartens in 2015 [18]. Additionally, school dental services and drinking water fluoridation are important universal tools for community based oral health promotion, and should be extended and continued. Myers-JDC-Brookdale Institute’s research on dental service utilization showed that dental check-ups provided by the school dental service increases uptake of dental care [19]. The effectiveness of appropriate fluoride levels in drinking water is widely documented. Its influence on Israeli dental health was recently demonstrated in Hadassah research [20].

## Conclusions

The level of dental disease among Israeli children has remained rather constant. However, after the reform, an increase in the treatment component was observed, compared to former studies.

In order to reduce caries prevalence, school dental services and drinking water fluoridation should be extended and continued. Primary preventative dental services should be established for children from birth with an emphasis on primary health care settings such as family health centers and kindergartens.

## Abbreviations

AHS, additional health services; dmft, decayed, missing and filled teeth index; HMO, Health Maintenance Organizations; LA, local authorities; NHIL, National health Insurance Law; NIHP, Israel National Institute for Health Policy; SDS, School Dental Service; SDS, School Dental Services; SES, socioeconomic strata
